# Lipid–Saporin Nanoparticles for the Intracellular Delivery of Cytotoxic Protein to Overcome ABC Transporter-Mediated Multidrug Resistance In Vitro and In Vivo

**DOI:** 10.3390/cancers12020498

**Published:** 2020-02-21

**Authors:** Guan-Nan Zhang, Pranav Gupta, Ming Wang, Anna Maria Barbuti, Charles R. Ashby, Yun-Kai Zhang, Leli Zeng, Qiaobing Xu, Ying-Fang Fan, Zhe-Sheng Chen

**Affiliations:** 1 Department of Pharmaceutical Sciences, College of Pharmacy and Health Sciences, St. John’s University, Queens, NY 11439, USA; guannan.zhang12@stjohns.edu (G.-N.Z.); pgupta0@mgh.harvard.edu (P.G.); anna.barbuti13@my.stjohns.edu (A.M.B.); yunkai.zhang12@stjohns.edu (Y.-K.Z.); zengleli0736@163.com (L.Z.); 2 Department of Biomedical Engineering, Tufts University, Medford, MA 02155, USA; mingwang214@gmail.com (M.W.); Qiaobing.Xu@tufts.edu (Q.X.); 3 Tomas Lindahl Nobel Laureate Laboratory, Research Centre, The Seventh Affiliated Hospital, Sun Yat-sen University, Shenzhen 518107, China; 4 Department of Hepatobiliary Surgery, Zhujiang Hospital, Southern Medical University, Guangzhou 510282, China

**Keywords:** cancer, multidrug resistance, ATP-binding cassette transporter, saporin, EC16-1, nanoparticles

## Abstract

Although the judicious use of anticancer drugs that target one or more receptor tyrosine kinases constitutes an effective strategy to attenuate tumor growth, drug resistance is commonly encountered in cancer patients. The ATP-binding cassette transporters are one of the major contributors to the development of multidrug resistance as their overexpression significantly decreases the intracellular concentration and thus, the efficacy of certain anticancer drugs. Therefore, the development of treatment strategies that would not be susceptible to efflux or excretion by specific ABC transporters could overcome resistance to treatment. Here, we investigated the anticancer efficacy of saporin, a ribosome-inactivating protein. Since saporin has poor permeability across the cell membrane, it was encapsulated in a lipid-based nanoparticle system (EC16-1) that effectively delivered the formulation (EC16-1/saporin) intracellularly and produced anti-cancer efficacy. EC16-1/saporin, at nanomolar concentrations, significantly inhibited the cellular proliferation of parental and ABCB1- and ABCG2-overexpressing cancer cells. EC16-1/saporin did not significantly alter the subcellular localization of ABCB1 and ABCG2. In addition, EC16-1/saporin induced apoptosis in parental and ABCB1- and ABCG2-overexpressing cancer cells. In a murine model system, EC16-1/saporin significantly inhibited the tumor growth in mice xenografted with parental and ABCB1- and ABCG2-overexpressing cancer cells. Our findings suggest that the EC16-1/saporin combination could potentially be a novel therapeutic treatment in patients with parental or ABCB1- and ABCG2-positive drug-resistant cancers.

## 1. Introduction

Multidrug resistance (MDR) is a phenomenon in which cancer cells develop resistance to structurally and mechanistically unrelated anticancer drugs [1]. MDR is one of the major causes of chemotherapy failure and increased cancer patient mortality. In the last decades of research into the cause of MDR, several mechanisms have been identified. However, the overexpression of the ATP-binding cassette (ABC) transporters in cancer continues to be the primary mediator of MDR. The ABC transporters are a family of proteins that utilize the energy derived from the hydrolysis of adenosine triphosphate (ATP) to adenosine diphosphate (ADP) [2,3,4]. The hydrolysis of ATP by the ABC transporters catalyzes the efflux of the substrate drugs from the cancer cells, reducing their intracellular drug concentration and their efficacy [5,6]. In normal cells, the ABC transporters prevent the intracellular accumulation of potentially toxic endogenous and xenobiotic compounds; however, in cancer cells, the overexpression of these transporters significantly reduces the intracellular concentration of the substrate anticancer drug, producing desensitization, and eventually, resistance to drugs with distinct structures and mechanisms of action. To date, 49 human ABC transporters have been identified, which have been further subdivided into 7 subfamilies (ABC A through G). The two ABC transporters most commonly involved with the development of MDR in cancer cells are the ABC transporter subfamily B member 1 (ABCB1/P-gp/MDR1) and the ABC transporter subfamily G member 2 (ABCG2/BCRP/MXR) [7,8,9,10]. These two transporters are structurally distinct in that the ABCB1 transporter contains two nucleotide-binding domains (NBDs) and two transmembrane-binding domains (TMDs) [11] whereas the ABCG2 transporter, also known as a “half transporter”, contains only one TMD and one NBD, which requires a dimerization step for functional transport activity. [12,13]

The ABCB1 transporter protein encoded by the ABCB1 gene located on chromosome 7p21 was the first identified ABC transporter [14,15]. The ABCB1 transporter is present on the apical membrane in a variety of tissues, including the blood–brain barrier, intestines, adrenal glands, placenta, and kidney. The transport function is vital for these tissues as it plays an important role in protecting cells from the accumulation of xenobiotics and cellular toxicants [16,17,18]. The ABCB1 transporter catalyzes the transport of a variety of classes of anticancer drugs, including taxanes (e.g., paclitaxel, doxetaxel), epipodophyllotoxins (e.g., etoposide and teniposide), vinca alkaloids (e.g., vinblastine and vincristine), and anthracyclines (e.g., doxorubicin and daunorubcin) [19,20,21]. The ABCG2 transporter is expressed in placental syncytiotrophoblasts, the apical surface of small intestines, colon epithelium, luminal surfaces of microvessels, endothelium of human brain, and in the veins and capillaries of blood vessels [22,23,24]. The expression of ABCG2 transporters in tumor tissues is associated with the development of resistance to chemotherapeutic drugs, such as nucleoside analogs, anthracyclines, camptothecin-derived topoisomerase I inhibitors, methotrexate, and flavopiridols [25,26]. Furthermore, clinical studies conducted in patients with adult and childhood leukemia indicate that the overexpression of the ABCG2 transporter is significantly correlated with a poor clinical prognosis [27,28].

Several recent studies have been conducted to investigate the potential of surmounting MDR by blocking the function of ABC transporters or designing novel anticancer compounds that can bypass the efflux function of these transporters. In the search for novel inhibitors of ABC transporters, the first- and second-generation tyrosine kinase inhibitors (TKIs) were considered unacceptable due to their pharmacokinetic interactions with other chemotherapeutic drugs. Although third-generation TKIs have less toxicity than the first two generations, the third-generation inhibitors did not produce beneficial results in clinical trials. Thus, it is pertinent to develop new strategies that are effective and safe while avoiding ABC transporter-mediated efflux, such as anticancer prodrugs, which can bypass adverse pharmacokinetic interactions as well as circumvent the ABC transporter function. Novel formulations can deliver anticancer drugs directly to the target site while safely bypassing the efflux transporters. One approach involves nanoparticle systems that encapsulate a protein-based toxin, allowing for a reduced concentration of anticancer drug compared to conventional small molecule-based drugs [29]. Certain nanoparticles can enter the cell by endocytosis across the cell membrane, and the loaded drug would be transported away from the ABC transporters [30,31]. This approach can be used to co-deliver an anticancer drug in combination with an inhibitor of the ABC transporters, in drug-resistant tumors, avoiding the efflux effect on the intracellular drug concentration and thus re-sensitizing the cancer cells to the anticancer drug.

Since the 1980s, protein therapy, such as insulin-based formulations, have been used as a safe and effective method to treat various diseases [32,33,34]. The majority of the protein-based drugs exert their therapeutic efficacy by binding to targets located on the cell surface or extracellular domains. Although studies have shown that the therapeutic benefits of protein-based drugs are greater than conventional drug delivery techniques, the delivery of protein drug intracellularly is still a considerable challenge. Saporin is a ribosome-inactivating protein (RIP) that was isolated from the seeds of *Saponaria officinalis* [35,36]. Saporin belongs to type I RIPs and consists of a single polypeptide chain, with a molecular weight of 30 KDa [37,38]. The high level of enzymatic activity and resistance to conjugation procedures and proteases makes *Saponaria officinalis* a potent toxin that produces anticancer efficacy by inducing apoptosis and the inhibition of the proliferation of cancer cells [39,40]. However, the use of saporin as an anticancer drug is limited by its poor penetration into cancer cells, thus reducing its intracellular concentration and efficacy. EC16-1 is a lipid-based nanoparticle formulation that loads saporin through electrostatic and hydrophobic interactions [41]. Previously, EC16-1 has been shown to deliver saporin intracellularly in a panel of cancer cell lines with high efficiency and significant anticancer efficacy [42,43]. In this study, we determined the anticancer efficacy of an EC16-1-loaded saporin combination (EC16-1/saporin) in drug-resistant cancer cells. Since the major EC16-1/saporin diffuses across the cell membrane by endocytosis, we conducted experiments to use cytotoxic proteins/drugs that are too large to be effluxed by the MDR efflux pumps, thus re-sensitizing the MDR tumors to anticancer drugs.

## 2. Results

### 2.1. Assessment of the Magnitude of Resistance in ABCB1- and ABCG2-Overexpressing Cells

**Figure 1:**
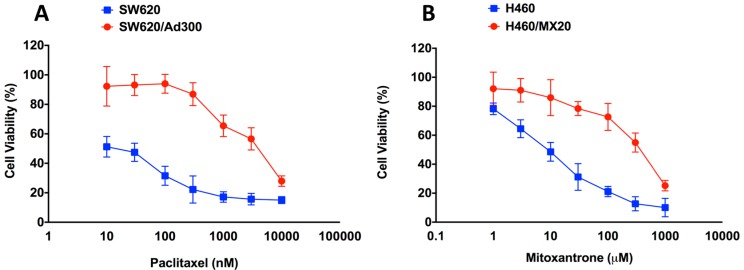
Cytotoxicity of paclitaxel or mitoxantrone in parental and ABCB1- or ABCG2-overexpressing cells. The MTT assay was conducted to determine the effect of paclitaxel on the viability of parental SW620 and ABCB1-overexpressing SW620/AD300 cells (**A**). The effect of mitoxantrone on the viability of parental NCI-H460 and ABCG2-overexpressing NCI-H460/MX20 cells (**B**). The points with error bars represent the mean ± SD for independent determinations in triplicate. The above figures are representative of three independent experiments.

Since our aim was to determine whether the expression of ABCB1 or ABCG2 affects the cytotoxicity of EC16-1/saporin, we assessed the resistant profiles of our ABCB1- and ABCG2-overexpressing cell lines. The MTT assay was used to assess resistance in the parental and resistant pairs, SW620 versus SW620/AD300, and NCI-H460 versus NCI-H460/MX20 cells. Our results indicated that paclitaxel (an ABCB1 substrate) had an IC50 value of 66.08 ± 5.81 nM in the SW620 cells and about 10,000 nM in the SW620/AD300 cells, thus yielding a resistance fold of about 80 (Figure 1A). Furthermore, mitoxantrone (an ABCG2 substrate) had an IC50 value of 12.4 ± 1.08 nM in the NCI-H460 cells and 990 ± 11.21 nM in the NCI-H460/MX20 cells, thus yielding a resistance fold of >150 (Figure 1B).

### 2.2. Effect of EC16-1/Saporin on the Cellular Proliferation of Parental and Resistant ABCB1- and ABCG2-Overexpressing Cells

**Figure 2:**
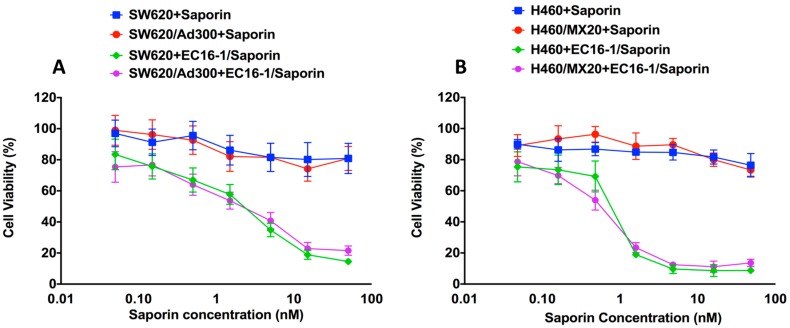
Cytotoxicity of saporin or EC16-1/Saporin in parental and ABCB1- or ABCG2-overexpressing cells. The MTT assay was used to determine the effect of saporin or EC16-1/saporin on the viability of parental SW620 and ABCB1-overexpressing SW620/AD300 cells after 72 h of incubation (**A**). Effect of saporin or EC16-1/saporin on the viability of parental NCI-H460 and ABCG2-overexpressing NCI-H460/MX20 cells (**B**). The lines represent the mean of triplicate determinations (*n* = 3). The error bars represent the SD.

Lipidoid EC16-1 was synthesized through using a ring-opening reaction between 1,2-epoxyhexadecane and N,N′-Dimethyl-1,3-propanediamine [41]. The MTT assay was used to determine the cytotoxicity of EC16-1 alone, saporin alone, or EC16-1/saporin, in the cell lines used in this study. As shown in Figure 2A, the IC50 value of saporin was not significantly different in the parental SW620 (IC50 = >50 nM) and ABCB1-overexpressing SW620/AD300 (IC50 = >50 nM) cells. However, EC16-1/saporin produced significant cytotoxicity in the SW620 and SW620/AD300 cells, based on the decrease in the IC50 value (3.44 ± 0.57 and 2.50 ± 0.32 nM, respectively). A similar effect was observed in the parental NCI-H460 and ABCG2-overexpressing NCI-H460/MX20 cells (Figure 2B). In these cells, saporin had IC50 values of >50 nM whereas, EC16-1/saporin had an IC50 value of 0.88 ± 0.03 nM and 0.63 ± 0.02 nM, respectively.

We also determined the effects of EC16-1 alone on the proliferation of the cell lines used in this study. EC16-1 produced a slightly higher but non-significant cytotoxicity compared to saporin (Table 1 and Table 2). These results clearly indicate that the EC16-1/saporin combination potently inhibits the cellular proliferation of parental, ABCB1, and ABCG2-overexpressing cells compared to saporin or EC16-1 alone.

### 2.3. Effect of EC16-1/Saporin on the Intracellular Localization of ABCB1 and ABCG2

**Figure 3:**
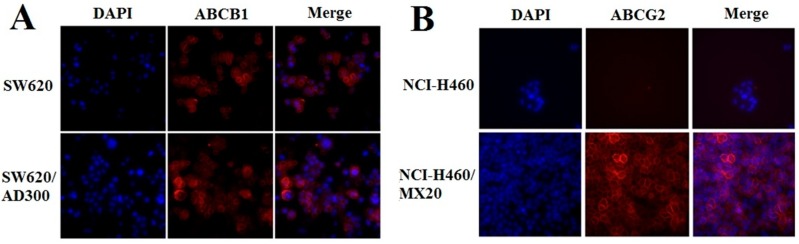
Effect of EC16-1/Saporin on the intracellular localization of ABCB1 or ABCG2. The parental and ABCB1- or ABCG2-overexpressing cells were treated with E16-1/saporin, at 5 nM, for 24h and stained with fluorescent ABCB1 or ABCG2 antibody, as described in the material and methods and observed under a fluorescent microscope. The effect of EC16-1/saporin on the intracellular localization of ABCB1 (**A**) or ABCG2 (**B**). DAPI (blue) counterstains the nuclei. Magnification, 20× (fluorescence microscopy).

Since ABCB1 and ABCG2 are membrane proteins, their translocation to the cytoplasm would diminish their efflux function. Therefore, EC16-1/saporin could decrease the intracellular levels by decreasing the translocation of the ABCB1 and ABCG2 transporter proteins into the cell membrane. We used an immunofluorescence assay to determine the presence of the ABCB1 and ABCG2 proteins in the parental and ABCB1- and ABCG2-overexpressing cells incubated with EC16-1/saporin. EC16-1/saporin (5 nM) did not significantly alter the localization and internalization of ABCB1 (Figure 3A) or ABCG2 (Figure 3B). These findings suggest that the cytotoxic effects of EC16-1/saporin on the ABCB1- or ABCG2-overexpressing cells were not due to an alteration in the cellular localization of these proteins.

### 2.4. Effect of EC16-1/Saporin on the Apoptosis of ABCB1- or ABCG2-Overexpressing Cells

**Figure 4:**
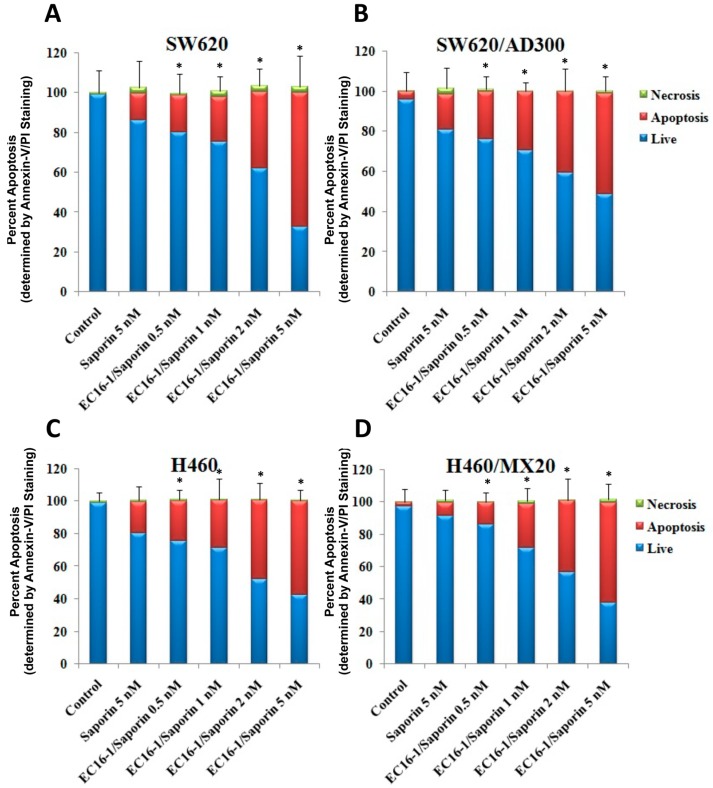
Effect of EC16-1/saporin on the induction of apoptosis in parental and ABCB1- or ABCG2-overexpressing cells. The parental SW620 (**A**) and ABCB1-overexpressing SW620/AD300 (**B**) cells were incubated with saporin (5 nM) or EC16-1/saporin. The parental NCI-H460 (**C**) and ABCG2-overexpressing NCI-H460/MX20 (**D**) cells were incubated with saporin (5 nM) or EC16-1/saporin. The apoptotic cell population was quantified using flow cytometry. The bar graphs represent the average cell population from three independent experiments and the error bars represent the SD. * *p* < 0.05 compared with control.

To determine if the inhibition of the growth of the parental and ABCB1- and ABCG2-overexpressing cancer cells by EC16-1/saporin was due to the induction of apoptosis, the parental and ABC-overexpressing cells were incubated with various concentrations of EC16-1/saporin for 24 h and apoptosis was determined as described in the methods. The majority of the cells were viable in the control group and showed minimal or no signs of apoptosis (Figure 4, blue color bars). After incubation with 5 nM of saporin, there was a non-significant increase in the number of apoptotic cells in SW620, SW620/AD300, H460, and H460/MX20. Interestingly, EC16-1/saporin produced a concentration-dependent increase in the number of apoptotic cells in all of the cell lines. EC16-1/saporin, at 5 nM, produced an increase in the apoptotic cell population in the parental SW620 (Figure 4A) and ABCB1-overexpressing SW620/AD300 cells (Figure 4B), compared to the control (0.98% vs. 68.70% and 5.83% vs. 53.21%, respectively). Similarly, EC16-1/saporin, at 5 nM, produced an increase in the apoptotic cell population in parental NCI-H460 (Figure 4C) and ABCG2-overexpressing NCI-H460/MX20 cells (Figure 4D), compared to the control (0.76% vs. 61.02% and 3.11% vs. 66.10%, respectively).

These results indicated that induction of apoptosis by EC16-1/saporin inhibits cancer cell proliferation in parental, ABCB1-, and ABCG2-overexpressing cancer cells.

### 2.5. EC16-1/Saporin Decreases Tumor Volume and Weight in an ABCB1-Overexpressing Tumor Xenograft Model

Based on in vitro findings, we subsequently conducted experiments to determine the efficacy of EC16-1/saporin in an in vivo mouse tumor xenograft model. Parental (SW620) and ABCB1-overexpressing drug-resistant (SW620/AD300) cells were implanted into athymic nude mice to create the ABCB1-overexpressing tumor xenograft model. 

**Figure 5:**
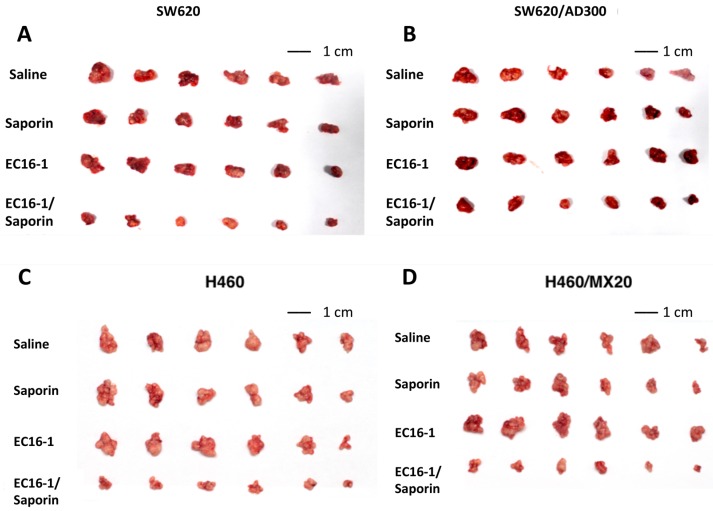
Effect of EC16-1/saporin on the parental and ABCB1 or ABCG2 tumors in nude athymic mice. The images of excised SW620 (**A**) and SW620/AD300 (**B**) tumors implanted subcutaneously in athymic NCR nude mice (*n* = 6). Images of the excised NCI-H460 and (**C**) and NCI-H460/MX20 (**D**) tumors implanted subcutaneously in athymic NCR nude mice (*n* = 6). The images were taken at the end of the 12-day treatment period.

**Figure 6:**
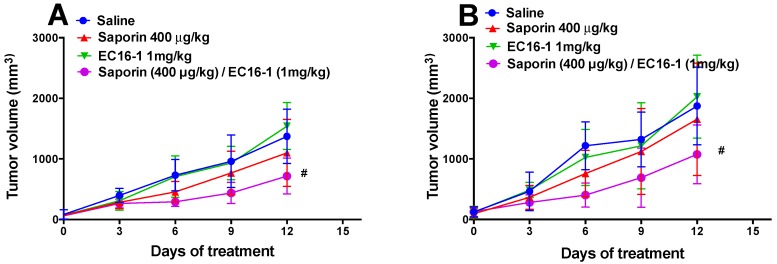

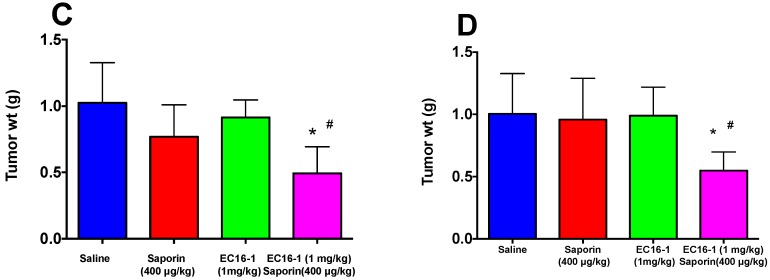
Effect of EC16-1/saporin on the volume and weight of SW620 and SW620/AD300 tumors. The changes in tumor volume over time in SW620 (**A**) and SW620/AD300 (**B**) in xenografts following implantation. Each point on the line graph represents the mean tumor volume (mm^3^) on each day after implantation. The error bars represent the SD. The mean weight (*n* = 6) of the excised SW620 (**C**) and SW620/AD300 (**D**) tumors from the mice treated with saline, saporin, EC16-1, and EC16-1/saporin, at the end of the 12-day treatment period. The error bars represent the SD. * *p* < 0.05, compared with the saporin alone group. ^#^ *p* < 0.05, compared with the control group.

As shown in Figure 5, there was a significant increase in the tumor volume in the SW620 (Figure 5A) and SW620/AD300 (Figure 5B) groups that were treated with saline. The administration of saporin (400 μg/kg, q3d × 4, i.p.) or EC16-1 (1 mg/kg, q3d × 4, i.p.) did not significantly reduce the tumor volume compared to animals treated with saline (Figure 5A,B and Figure 6A,B). However, EC16-1 (1 mg/kg)/saporin (400 μg/kg, q3d × 4) significantly reduced tumor growth compared to animals treated with saline. There was a significant reduction in tumor volume in both SW620 and SW620/AD300 tumors in animals treated with EC16-1/saporin, compared to the control, saporin, and EC16-1 groups. This effect on the tumor volume was first observed on day 3 and continued until day 12 (final day) of the treatment regimen. Furthermore, on the final day, we measured the weight of each tumor in the animals. As shown in Figure 6C,D, there was significant reduction in the weight of the tumors in the EC16-1/saporin group, compared to the control, saporin, and EC16-1 groups, for both SW620 and SW620/AD300 tumors.

### 2.6. EC16-1/Saporin Decreases Tumor Volume and Weight in ABCG2-Overexpressing Tumor Xenograft Models

In order to investigate the effect of EC16-1/saporin on the ABCG2-overexpressing tumors, the parental (NCI-H460) and ABCG2-overexpressing (NCI-H460/MX20) cells were implanted into athymic nude mice to create the ABCG2-overexpressing tumor xenograft model. 

**Figure 7:**
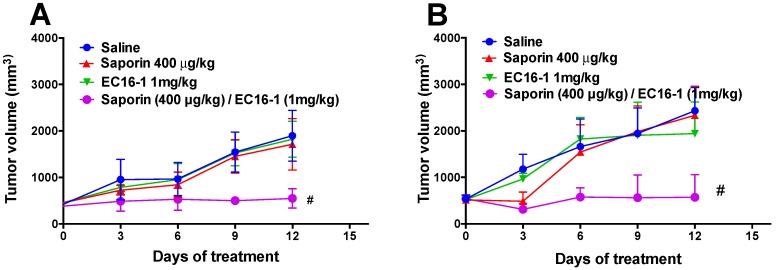

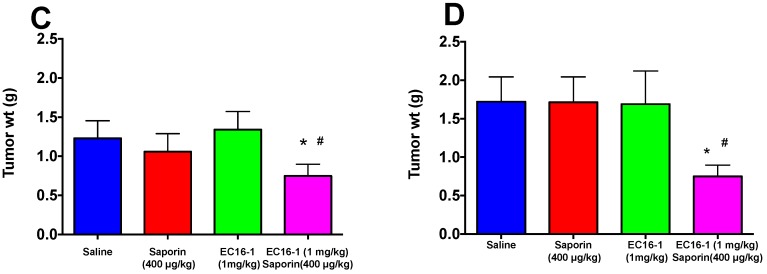
Effect of EC16-1/saporin on the tumor volume and weight of NCI-H460 and NCI-H460/MX20 tumors. The changes in tumor volume over time in NCI-H460 (**A**) and NCI-H460/MX20 (**B**) xenografts following tumor implantation. Each point on the line graph represents the mean tumor volume (mm3) on each day after implantation. The mean weight (*n* = 6) of the excised NCI-H460 (**C**) and NCI-H460/MX20 (**D**) tumors from the mice treated with saline, saporin, EC16-1, and EC16-1/saporin, at the end of the 12-day treatment period. The error bars represent the SD. * *p* < 0.05, compared to the saporin alone group. ^#^ *p* < 0.05, compared with the control group.

Similar to the SW620 and SW620/AD300 tumors, there was a significant increase in the tumor volume in the NCI-H460 (Figure 5C and Figure 7A) and NCI-H460/MX20 (Figure 5D and Figure 7B) tumors in animals treated with saline. There was a significant reduction in the tumor volume in animals treated with EC16-1/saporin, compared to the control, saporin, or EC16-1 groups, in both NCI-H460 and NCI-H460/MX20 tumors. This effect on the tumor volume was first observed on day 3 and continued until day 12 (final day) of the treatment regimen. In addition, we measured the effect of EC16-1/saporin on the weight of the tumors. EC16-1/saporin significantly decreased the tumor weight in both NCI-H460 and NCI-H460/MX20 tumors, compared to the control, saporin, or EC16-1 groups (Figure 7C,D).

### 2.7. Assessment of Potential Toxicity of EC16-1/Saporin

**Figure 8:**
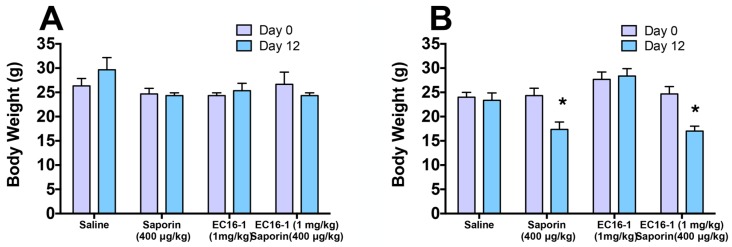
Effect of EC16-1/saporin on the body weight of animals bearing parental and ABCB1 or ABCG2 tumors. The changes in the mean body weight of mice (*n* = 6) bearing SW620 and SW620/AD300 tumors (**A**) and the changes in the mean body weight of mice (*n* = 6) bearing NCI-H460 and NCI-H460/MX20 tumors (**B**). The changes in the body weight are plotted for day 0 (before) and day 12 (after) of the treatment. The bar graphs represent the mean body weight and the error bars represent the SD. * *p* < 0.05 compared with day 0.

Given that toxicity is a major concern for any chemotherapeutic regimen, we regularly measured the body weights of each animal as a general indicator of their overall health throughout the course of the study. The body weight of each animal was recorded every three days, beginning on day 0 and ending on day 12 (final day). As shown in Figure 8A, there was no significant change in the body weight of the animals in the ABCB1 tumor xenograft models. These results indicated that EC16-1/saporin produces anti-cancer efficacy in parental and ABCB1-overexpressing tumors without producing any overt toxicity or weight loss. However, the animal body weight of the animals was significantly decreased by the EC16-1/saporin treatment compared with day 1, in the ABCG2 tumor xenograft models (Figure 8B).

## 3. Discussion

Following the approval of imatinib by the United States Food and Drug Administration in 2001, several anti-cancer drugs have been developed for the treatment of both benign and malignant tumors [44,45]. The majority of these drugs produce cytotoxic effects on tumor cells with minimal adverse effects in normal cellular physiology [46,47]. The discovery of novel pathways and mechanisms causing cancer has facilitated the development of targeted compounds that attenuate tumor growth with minimal toxicity.

Despite the advances in pharmacotherapeutics and translational medicine, the vast majority of patients fail to respond to conventional treatment strategies. This leads to the development of drug resistance and an increase in the overall mortality associated with cancer. Novel drug delivery techniques, such as encapsulation of a chemotherapeutic drug in a nano-carrier, has yielded significant success in improving the survival of cancer patients. Nanoparticle formulations have reduced particle size (in the nanometer range) and can be designed to deliver multiple drugs simultaneously [48]. Studies have reported that nanoparticles, as small as 100 nm, can easily penetrate the cell membrane through active endocytosis and reach the tumor tissue [49]. Anticancer nanoparticle drug formulations can deliver one or more chemotherapeutic drug (such as paclitaxel or doxorubicin) by encapsulating them in a protective coating. This protective layer can be a polymer, such as polyethylene glycol (PEG), which delivers the drug to the cellular target site, without being subjected to metabolism or degradation [50].

Nanoparticles offer a number of advantages over conventional drug delivery techniques, which includes: (1) Greater retention in the blood due to the reduced particle size; (2) easier delivery of drugs that have poor solubility and stability; (3) decreased drug clearance and increased bioavailability; and (4) a multi-functional and multi-targeted drug delivering platform [51,52,53]. Owing to their aforementioned advantages compared to conventional drug delivery systems, nanoparticles have also been developed to treat resistance to chemotherapeutic drugs. An increasing number of synthetic nanoparticles have been designed for this purpose, such as liposomes, polymeric nanoparticles, polymeric micelles, and dendrimers. PEGylate liposome (Doxil) liposomes loaded with doxorubicin and non-PEGylated liposome (Myocet) are clinically used to treat certain types of cancers. In addition, lipoplatin is a promising liposomal platinum drug formulation that is currently undergoing clinical investigation [54,55,56].

Recently, Wang and colleagues reported that a tea–nanoparticle system effectively delivered doxorubicin and overcame ABC transporter-mediated drug resistance by increasing the intracellular concentration of doxorubicin [57]. This system produced a greater drug accumulation and reversal of in vitro and in vivo MDR in a drug-resistant cancer model compared to conventional doxorubicin therapy. 

Saporin, a potent cytotoxic compound, has been shown to have anti-cancer efficacy. However, its use in anticancer treatment regimens has been limited due to its poor diffusion across the cell membrane. A strategy to kill MDR cells is to use cytotoxic proteins/drugs that are too large to be the ABC efflux transporters. Here, we determined the in vitro and in vivo efficacy of saporin encapsulated in a lipid-based system, EC16-1. Previous studies have shown that the combination EC16-1 and saporin has an enhanced entry into various cancer cell lines as compared to saporin [41,43]. We conducted experiments to determine the anti-cancer efficacy of EC16-1/saporin and its interaction with the ABC transporters ABCB1 and ABCG2. To our knowledge, this is the first study to report the in vitro and in vivo anti-cancer efficacy of EC16-1/saporin on parental and ABCB1- and ABCG2-overexpressing cells and in mouse xenograft tumor models.

Our results indicated that the ABCB1-overexpressing SW620/AD300 cells were greater than 80-fold resistant, as compared to the parental SW620 cells, whereas the ABCG2-overexpressing NCI-H460/MX20 cells were greater than 150-fold resistant, compared to the non-resistant parental NCI-H460 cells. As reported previously, the resistance fold (RF) was calculated by dividing the substrate drug IC50 values in the drug-resistant cells by the IC50 values in the parental cells [10,46,58]. The resistant profile of our cells was comparable to the previously published studies [12,59]. Our results indicated that our parental ABCB1- and ABCG2-overexpressing cells were an acceptable model to evaluate the anti-cancer and reversal efficacy of EC16-1/saporin.

We also investigated the efficacy of EC16-1/saporin on the proliferation of parental and ABCB1- or ABCG2-overexpressing cells. Our results indicated that the EC16-1/saporin combination produced significantly greater cytotoxicity compared to saporin alone. Since a significant decrease in the IC50 value of EC16-1/saporin was observed in both parental and ABCB1- or ABCG2-overexpressing cells, it can be concluded that the cytotoxic efficacy of EC16-1/saporin is not significantly affected by the presence of the ABCB1 or ABCG2 transporters. However, because the localization of ABC transporters is a major factor that regulates their efflux function, we determined the effect of EC16-1/saporin on the intracellular localization of the ABCB1 or ABCG2 transporters. The incubation of cells with EC16-1/saporin, at 5 nM, for 24 h, did not significantly alter the intracellular localization of the ABCB1 or ABCG2 transporters. Indeed, the expression of the ABCB1 and ABCG2 transporters was almost exclusively localized on the cell membrane. These results are in accordance with earlier studies, where chemotherapeutic-based nanoparticle formulations were reported to have no significant effect on the in vitro intracellular localization of ABC transporters [57,60].

Since our cell proliferation studies confirmed that EC16-1/saporin is a cytotoxic compound in the cancer cell types used in the study, we subsequently determined the effect of EC16-1/saporin on the induction of apoptosis in the parental and ABCB1- and ABCG2-overexpressing cells. Our results indicated that EC16-1/saporin induced apoptosis in parental and ABCB1- or ABCG2-overexpressing cells. Moreover, a concentration-dependent induction in apoptosis was observed following incubation with EC16-1/saporin for 24 h. These findings suggested that the cytotoxic effects of EC16-1/saporin in the parental and the ABCB1- and ABCG2-overexpressing cells are due to an induction of apoptosis. This finding is similar to previous studies indicating that certain saporin can produce cytotoxicity by inducing apoptosis [61,62]. 

The findings based on our in vitro experiments led us to determine the effect of EC16-1/saporin in the parental and the ABCB1- and ABCG2-overexpressing cancer cells using mouse xenograft tumor models. As previously reported, we established ABCB1- or ABCG2-overexpressing tumor xenograft models, with a slight modification [13,58]. The animals treated with saline (control group) had a significant increase in tumor growth over the 12-day treatment period. The saporin or EC16-1 alone treatment groups did not significantly attenuate tumor growth compared to the control group. However, the EC16-1/saporin combination treatment significantly inhibited tumor growth. A significant reduction in tumor volume and weight occurred in the parental and ABCB1- or ABCG2-overexpressing tumors, compared to the single treatment groups. Furthermore, EC16-1/saporin produced significant weight loss during the study period. The safety profile of EC16-1/saporin has not been determined in humans.

## 4. Materials and Methods

### 4.1. Reagents

Dulbecco’s modified eagle’s medium (DMEM), fetal bovine serum (FBS), penicillin/streptomycin, and trypsin 0.25% were purchased from Hyclone (Pittsburgh, PA, USA). Monoclonal antibody against ABCB1 and ABCG2 were purchased from Thermo Fisher Scientific Inc. (Rockford, IL, USA). Alexa Fluor 488 conjugated goat anti-mouse IgG secondary antibody was also purchased from Thermo Fisher Scientific Inc. Paclitaxel and mitoxantrone were purchased from Sigma-Aldrich (St. Louis, MO, USA). 3-(4,5-dimethylthiazol-2-yl)-2,5-diphenyl-tetrazolium bromide (MTT), and dimethyl sulfoxide (DMSO) were obtained from Sigma Chemical Co. (St. Louis, MO, USA). Propidium iodide (PI) and Annexin-V were purchased from BD biosciences (San Jose, CA, USA). 

### 4.2. EC16-1/Saporin Formulation Preparation

The synthetic lipid EC16-1 and lipid/saporin formulation were prepared according to our published procedure [41]. In brief, the EC16-1 was synthesized using a ring-opening reaction between 1,2-epoxyhexadecane and N,N′-Dimethyl-1,3-propanediamine. The reactants were heated at molar ratios of 1:2.4 without any solvent for 48 h, and purified using flash chromatography on silica gel. For in vitro experiment, EC16-1 was directly used for saporin complexation and delivery. For in vivo formulation, EC16-1/saporin nanoparticles were formulated by a thin film hydration method. Briefly, EC16-1, cholesterol, and DOPE were mixed at a weight ratio of 16:4:1 in chloroform, and the organic solvent was evaporated under vacuum. To formulate EC16-1/protein nanoparticles, thin layer film that had formed was re-hydrated with PBS to make a cloudy solution. The solution was then mixed with saporin at a weight/weight ratio of 2.5:1 (EC16-1:saporin). The EC16-1/protein complex was post-modified with mPEG2000-ceramide C16 (25% weight percentage in total compared to EC16-1), followed by a further 60 min of incubation at room temperature. The final EC16-1 concentration in all formulations was kept at 1 mg/mL.

### 4.3. Cell Lines and Cell Culture

The human colorectal adenocarcinoma cell line, SW620, and the doxorubicin-induced SW620/AD300-resistant cell line (overexpressing ABCB1) were used in this study. In addition, the non-small cell lung cancer cell line NCI-H460 and mitoxantrone-induced NCI-H460/MX20-resistant cell line (overexpressing ABCG2) were also used. The cells were cultured in DMEM media supplemented with 10% FBS and 1% penicillin/streptomycin at 37 °C in a humidified atmosphere of 5% CO_2_. The cells were grown in culture flasks as monolayers in drug-free medium for at least more than 2 weeks before the performed assays.

### 4.4. MTT Assay

The anti-proliferative efficacy of EC16-1, saporin, or the EC16-1/saporin combination were determined by a modified 3-(4,5-dimethylthiazol-2-yl)-2,5-diphenyl-tetrazolium bromide (MTT) colorimetric assay. Briefly, the cells were seeded onto 96-well plates at a density of 4 × 10^3^ cells per well. Following 24 h of incubation, the cells were incubated with EC16-1, saporin, or EC16/saporin at the indicated concentrations. After 72 h, 20 μL of MTT (4 mg/mL) was added to each well and the plates were further incubated at 37 °C for 4 h. Subsequently, the MTT–media solution was removed from each well and 100 μL of dimethyl sulfoxide (DMSO) was added to dissolve the formazan crystals formed by the mitochondrial reduction of MTT in proliferating cells. The absorbance values were then measured at 570 nm using an Opsys microplate reader (Dynex Technologies, Chantilly, VA, USA).

### 4.5. Immunofluorescence

Cells (3 × 10^4^) were seeded in 24-well plates and incubated overnight, followed by incubation with 5 nM of EC16-1/saporin for 24 h. After 24 h, the cells were fixed in 4% paraformaldehyde for 15 min and permeabilized by 0.1% Triton X-100 for 10 min. Subsequently, blocking was done with 6% BSA for 1.5 h at room temperature. The cells were incubated with monoclonal antibody C219 against ABCB1 (1:400) and BXP-21 against ABCG2 (1:400) overnight, followed by Alexa Fluor 488 conjugated secondary antibody (1:1000) for 1 h. DAPI was used to counterstain the nuclei. Immunofluorescence images were collected using a Nikon TE-2000S fluorescence microscope (Nikon Instruments Inc., Melville, NY, USA).

### 4.6. Apoptosis Assay

The cells were incubated with either saporin alone at 5 nM or EC16-1/saporin at 0.5, 1, 2, and 5 nM for 24 h. After 24 h, the cells were collected and washed twice with PBS supplemented with 0.5% bovine serum albumin (BSA) at 4 °C. The cell pellet was fixed overnight in ice cold 70% ethanol at 4 °C. The cells were subsequently stained with FITC-labeled Annexin-V (BD Pharminogen, San Diego, CA, USA) and PI, at 37 °C for 30 min. Flow cytometric analysis was performed to determine the apoptotic cell population.

### 4.7. Experimental Animals

Four- to six-week-old male athymic NCR (nu/nu) nude mice (homozygous, albino color), weighing 18–22 g, were used for the ABCB1 or ABCG2 xenograft models. These animals were purchased from Taconic Farms (Taconic Biosciences, New York, NY, USA). All animals were provided with sterile water, rodent chow ad libitum, and maintained on an alternating 12-h light/dark cycle. All animals were housed at the animal facility of St. John’s University. All animals were treated humanely in accordance with the guidelines set forth by the American Association for Accreditation of Laboratory Animal Care and the U.S. Public Health Service Policy on Humane Care and Use of Laboratory Animals. All experiments were approved by the Institutional Animal Care and Use Committee (IACUC) at St. John’s University (Queens, NY, USA) and carried out in accordance with the guidelines on animal care and experiments of laboratory animals (animal study protocol number: 1842).

### 4.8. Methodology for Preclinical Antitumor Efficacy

Briefly, matching cell line pairs, parental and resistant, were implanted into the left and right armpit region, respectively, of each mouse. The parental SW620 (6 × 10^6^) and ABCB1-overexpressing SW620/AD300 (6 × 10^6^) cells were injected subcutaneously into one set of mice. The parental NCI-H460 (5 × 10^6^) and ABCG2-overexpressing NCI-H460/MX20 (5 × 10^6^) cells were injected subcutaneously into the second set of mice. Once the tumors reached a mean diameter of 0.5 cm, the mice were randomly dived into four treatment groups (*n* = 6) and administered either: (a) Vehicle (normal saline, q3d × 4, i.v.); (b) saporin (400 μg/kg, q3d × 4, i.v.); (c) EC16-1 (1 mg/kg, q3d × 4, i.v.); and (d) EC16-1 (1 mg/kg)/saporin (400 μg/kg) (q3d × 4, i.v.). The doses selected for saporin and EC16-1 were non-toxic to the animals and were based on the results of our pilot study. The animals were monitored carefully, and tumor sizes were measured and recorded using a caliper. The body weight of animals was recorded every 3rd day to access any drug-related toxicities as well as disease progression. The tumor volume was calculated by using the following formula: V = π/6 ((A+B)/2)^3^, where A and B are the two perpendicular diameters of a tumor. At the end of the study, all the animals were euthanized using carbon dioxide. The tumor tissues were excised, weighed, and stored at −80 °C until further analysis.

### 4.9. Statistical Analysis

All the experiments were repeated at least three times and the differences were determined using the two-tailed Student t-test. The a priori significance level was *p* < 0.05.

## 5. Conclusions

Our results indicated that saporin, a potent cytotoxic compound, when encapsulated in the lipid-based EC16-1 nanoparticle, significantly inhibits the proliferation of cancer cells. The EC16-1/saporin complex induces apoptosis in the parental cancer cells and in the resistant ABCB1- and ABCG2-overexpressing cells and does not significantly alter the intracellular localization of ABCB1 or ABCG2. Finally, EC16-1/saporin significantly decreases the growth of the parental and ABC transport-mediated resistant tumors in vivo, based on our tumor xenograft mouse models. Therefore, the EC16-1/saporin nanocomplex represents a novel anticancer strategy, not only for cancer patients with non-resistant tumor but also for patients with ABCB1 or ABCG2 drug-resistant cancers.

## Figures and Tables

**Table 1:** Effect of EC16-1/saporin on the cellular proliferation of SW620 and SW620/AD300 cells.

	SW620			SW620/AD300		
	EC16-1	Saporin	EC16-1/Saporin	EC16-1	Saporin	EC16-1/Saporin
IC50 ^a^	>50	>50	3.44 ± 0.63	34.45 ± 2.92	>50	2.50 ± 0.43
RF ^b^	1.00	1.00	1.00	0.69	1.00	0.74

^a^ Cytotoxicity of EC16-1, Saporin, and EC16-1/Saporin in parental SW620 and ABCB1 overexpressing. SW620/AD300 cell lines; an IC50 value are represented as the mean ± SD of three independent experiments performed in triplicate. ^b^ Values represent the resistance fold (RF), which was obtained by dividing IC50 values of resistant cell lines by those of the parental cell lines.

**Table 2:** Effect of EC16-1/saporin on the cellular proliferation of NCI-H460 and NCI-H460/MX20 cells.

	NCI-H460			NCI-H460/MX20		
	EC16-1	Saporin	EC16-1/Saporin	EC16-1	Saporin	EC16-1/Saporin
IC50 ^a^	19.57 ± 0.93	>50	0.88 ± 0.12	17.51 ± 1.32	>50	0.63 ± 0.07
RF ^b^	1.00	1.00	1.00	0.89	1.00	0.71

^a^ Cytotoxicity of EC16-1, Saporin, and EC16-1/Saporin in parental NCI-H460 and ABCG2 overexpressing NCI-H460/MX20 cell lines. An IC50 value is represented as the mean ± SD of three independent experiments performed in triplicate. ^b^ Values represent the resistance fold (RF), which was obtained by dividing IC50 values of resistant cell lines by those of the parental cell lines.
